# Synthesis of Amides from Amines and Esters Under Hydrothermal Conditions

**DOI:** 10.1002/open.202500508

**Published:** 2026-02-05

**Authors:** Prince Antwi Brown, Alexandria Aspin, Ziming Yang

**Affiliations:** ^1^ Department of Chemistry Oakland University Rochester Michigan USA

**Keywords:** amides, amines, esters, metal salts, pH

## Abstract

Organic synthesis under hydrothermal conditions provides a green and environmentally friendly method that can minimize waste and avoid toxic byproducts. In this study, we investigate ester aminolysis in hydrothermal water at 250°C and *P*_sat_. Among the studied substrates, ethyl acetate with benzylamine yields the highest amide concentration, followed by ethyl acetate with cyclohexylamine and ethyl benzoate with benzylamine. Time‐series experiments reveal that a dominating pathway initiates with hydrolysis of ester to form carboxylic acid, followed by the condensation between the acid and amine. The reaction proceeds more efficiently under neutral and basic than acidic conditions, suggesting the protonation of amines at lower pH inhibits the amide formation. The effects of common metal salts, such as NaCl, FeCl_3_, FeCl_2_, CuCl_2_, and ZnCl_2_, on amide hydrothermal synthesis are also studied, in which all tested metal salts show an inhibition on the amide yield. In the phosphate‐buffered experiments, however, most of the metal salts show an increase in amide formation compared to the non‐buffered experiments, suggesting the inhibition from the metal salts is caused by the decrease of pH in dissolved metal solutions. These findings suggest another feasible synthetic pathway of amides under hydrothermal conditions, which is subject to the solution pH and complexation with metal ions.

## Graphical abstract

In this study, we explore the amide synthesis under hydrothermal conditions, using amines and esters as the starting materials. Proposed major pathway starts with ester hydrolysis, followed by condensation between carboxylic acids and amines. The amide yield is dependent on the substrate structure, solution pH, and the presence of dissolved metal salts.

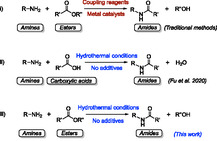

## 1 Introduction

Amides are one of the most important organic functional groups with many applications in medicine, material science, and biological systems [1, 2]. For example, frovatriptan and axitinib are amide‐containing medicines used to treat migraine and kidney cancer, respectively [3]. In the materials industry, polyamides produced from amides provide materials with high tensile strength and heat resistance, thereby enhancing their durability [2, 4]. Synthesis of peptides and proteins from amino acids through both biological and abiotic processes is also considered a key process for producing and sustaining life in terrestrial and oceanic habitats [5, 6].

**FIGURE 1:**
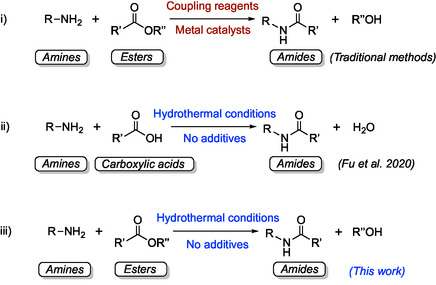
(i) Synthesis of amides between amines and esters using traditional methods, (ii) hydrothermal synthesis of amides between amines and carboxylic acids without additives (Fu et al., 2020 [8]), and (iii) hydrothermal synthesis of amides between amines and esters without additives (this work).

Biologically, amides are synthesized through enzymatic reactions involving proteases, amidases, and hydrolases, or ribosomal synthesis [7]. Traditional laboratory amide synthesis forms a carbon–nitrogen (C—N) bond when an electrophilic acyl group combines with nucleophilic nitrogen‐containing compounds in the presence of a coupling agent (Figure 1) [3, 9]. Other amide synthesis methods involve the production of a carbon–oxygen (C—O) bond by oxidation and hydration processes [7]. However, these methods use catalysts and coupling reagents that could be expensive, generate large amounts of waste, and yield toxic byproducts, which could be detrimental to human health and the environment [10]. Because of this, there is a growing need for green and sustainable alternatives.

In recent years, hydrothermal synthesis has emerged as a promising green synthetic route for the formation of a wide range of organic molecules [11, 12, 13]. As temperature and pressure increase, the dielectric constant of water decreases and its dissociation constant increases. The lower dielectric constant of water enables it to behave as an organic solvent that more readily dissolves hydrophobic organic compounds [14]. The elevated dissociation constant results in higher concentrations of hydronium and hydroxide ions, thus allowing water to act as an acid or base to catalyze organic reactions [15, 16]. Hydrothermal water has also been used as a green alternative in the organic synthesis of complex compounds such as bisimides [17], quinoxalines [18], and benzimidazoles [19], eliminating the need for organic solvents, strong acids, and toxic catalysts that are commonly required for traditional synthetic routes.

Previous experimental and theoretical studies have shown that amides can be synthesized by coupling acyl groups and amines under hydrothermal conditions (Figure 1). For example, Rushi and Simonet synthesized amides from nonadecanoic acid and ammonium bicarbonate with water heated at 300°C for 72 h [20], while Fu et al. synthesized amides from carboxylic acid and amine in pure water at 250°C within hours, achieving yields of up to 90% without any catalysts [8]. Geochemical models have also been developed to estimate and quantify amide bond formation in hydrothermal systems [21]. In addition, a previous study on the effect of metal salts on the hydrothermal stability of amides also indicated that amide synthesis could be sensitive to fluid composition and geochemical surroundings [22].

To the best of our knowledge, the aminolysis of esters (i.e., reaction of esters with amines) under hydrothermal conditions has been one of the least studied routes for amide synthesis. Most direct ester‐to‐amide synthetic reactions follow the conventional methods that utilize coupling reagents and metal catalysts such as EDC (1‐ethyl‐3‐(3‐dimethylaminopropyl)carbodiimide) and DCC (N,N′‐dicyclohexylcarbodiimide) (Figure 1), which are not cost‐effective or environmentally friendly [23, 24]. To seek a more sustainable and green approach, this study aims to investigate amide formation between amines and esters in hydrothermal fluids, where water replaces traditional organic solvents without the addition of toxic and expensive catalysts. The goals of this study are (1) to investigate the scope of the reaction using alkyl and aryl esters with primary, secondary, and aryl amines and (2) to examine the effects of pH and Earth‐abundant metal salts on the reaction. The proposed method would also contribute to sustainability in chemistry and industry by providing an alternative synthetic method for amides that focuses on reducing waste and hazardous byproducts that could impact the environment and human health.

## 2 Experimental Section

### 2.1 Materials

Ethyl acetate (99.9%), ethyl benzoate (99%), benzylamine (99%), cyclohexylamine (≥99.9%), and diphenylamine (99%) were purchased from Sigma‐Aldrich and used as the starting materials for the reaction. Ethyl benzoate and ethyl acetate were selected as representative esters (aryl and alkyl, respectively), along with benzylamine (primary aryl amine), cyclohexylamine (primary alkyl amine), and diphenylamine (secondary amine). These reactants were chosen to gain mechanistic insight into the various types of structures and because the aromatic rings of these structures can be easily detected using gas chromatography (GC). N‐benzylacetamide (99%, Chem‐Impex Inc.) and N‐benzylbenzamide (98%, Sigma‐Aldrich) were used as analytical standards to quantify the amide products on the GC. Dichloromethane (DCM, ≥99.9%, VWR) was used as the organic extraction solvent, and dodecane (≥98%, Sigma‐Aldrich) was used as an internal standard for GC analysis. Hydrochloric acid (HCl, 50%v/v, VWR) and sodium hydroxide (NaOH, >98%, Sigma‐Aldrich) were used for the pH experiments. Sodium chloride (NaCl, 99%, Sigma‐Aldrich), zinc chloride (ZnCl_2_, 97%, Alfa Aesar), ferrous chloride (FeCl_2_, 98%, Sigma‐Aldrich), ferric chloride anhydrous (FeCl_3_, 98%, Alfa Aesar), and cupric chloride (CuCl_2_, 99%, Sigma‐Aldrich) were used in the metal salts experiments. Sodium phosphate monobasic monohydrate (98%, Sigma‐Aldrich) and sodium phosphate monobasic dihydrate (99%, Sigma‐Aldrich) were used to adjust and buffer the pH of hydrothermal solutions.

### 2.2 Methods

The hydrothermal experiments were conducted following previously developed methods [25, 26, 27]. Briefly, the selected amine and ester were loaded in a fused silica glass tube (6 mm OD × 2 mm ID, GM Associates) with starting concentrations of 0.1 and 0.2 molal, respectively. Deionized water (DI, 18.2 MΩ cm) or a metal salt solution (0.1 molal NaCl, FeCl_3_, FeCl_2_, CuCl_2_, or ZnCl_2_) was added to the tubes using a gas‐tight syringe. Oxygen was removed from the tubes by three freeze–thaw cycles under vacuum using a pump and sealed with an oxyhydrogen flame torch to ensure a closed environment. The sealed tubes were then placed inside a stainless‐steel tube and put in a preheated GC oven, where they were subjected to hydrothermal treatment at 250°C and *P*_sat_ (~40 bar) for durations of 6 or 24 h. After completion, the tubes were quenched in a water bath to stop the reaction.

Additional hydrothermal experiments were performed to examine the effect of the solution pH on amide synthesis. The solution pH was adjusted to pH 2.1 and 9.4 (in situ pH, as calculated using EQ 3/6) using HCl or NaOH, mimicking acidic and basic conditions. A phosphate buffer solution (0.5 molal) of pH 5.6 was prepared and used to ensure the pH remained near neutral under the conditions studied. Because the sealed hydrothermal reactors could not be probed during heating, in situ measurements of pH and metal speciation were not possible; instead, these parameters were estimated using thermodynamic calculations. EQ 3/6 was used to calculate the necessary pH of the buffer at room temperature and the corresponding ratios of phosphate salts needed to prepare the buffer [28]. The pH of all the solutions (including metal salt solutions) at room temperature (22°C) was measured using a Mettler Toledo S210 pH meter.

Following the hydrothermal experiments, the solutions were extracted using previously developed methods [29, 30, 31]. Briefly, the reaction products were collected using DCM containing 8.8 mM dodecane as an internal standard. To ensure full collection of amines and amide products, 60 µL of 4 M NaOH was added to the extracted samples, which were then vortexed and centrifuged at 3000 rpm for 3 min. The organic phase was then carefully separated from the aqueous phase and transferred to a clean glass vial for analysis using GC with a flame ionization detector (Agilent 7820A) and GC‐mass spectrometry (Agilent 7890A/5975C) to determine the amide yields and products, respectively. Calibration curves were constructed using the analytical standards of intermediate compounds on GC for the quantification of reaction yields.

## 3 Results and Discussion

**FIGURE 2:**
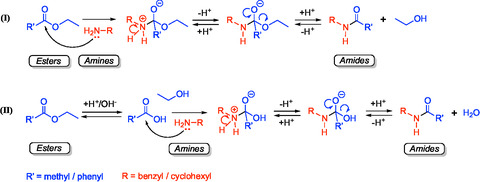
Proposed reaction pathways and mechanisms for amide synthesis from esters and amines as the starting materials, either via direct ester aminolysis (I) or via hydrolysis of esters to form carboxylic acids, followed by the condensation with amines (II).

The formation of amides from esters and amines is expected to proceed through a nucleophilic acyl substitution mechanism (Figure 2). In this pathway, amines act as nucleophiles, attacking the carbonyl carbon of the ester to form a tetrahedral intermediate. The intermediate further undergoes the elimination of an alkoxide group to yield the amide product. An alternative pathway involves the formation of carboxylic acid or carboxylate through the hydrolysis of esters (acid‐ or base‐catalyzed) under hydrothermal conditions (Figure 2), followed by the condensation reaction between carboxylic acids and amines, which has been previously reported by Fu et al., [8]. Both proposed pathways suggest that amide synthesis depends on the nature of the reactants (e.g., electrophilicity of the acyl group and nucleophilicity of the amine). To investigate the hydrothermal aminolysis of esters, three amines (benzylamine, cyclohexylamine, and diphenylamine) and two esters (ethyl acetate and ethyl benzoate) with varying structures and reactivities were selected for study. Among all the possible combinations of amines and esters, three reactions produced detectable amounts of amides under the experimental conditions (250°C and 24 h), including benzylamine with ethyl acetate (30.9% yield), benzylamine with ethyl benzoate (9.4% yield), and cyclohexylamine with ethyl acetate (14.2% yield) (Table S1). The identities of the observed amides were confirmed by GC‐MS and NMR spectroscopy (Figures S1–S5) as N‐benzylacetamide, N‐benzylbenzamide, and N‐cyclohexylacetamide, respectively.

The differences in amide yields are controlled by the steric accessibility and the effective nucleophilicity of the reactants under hydrothermal conditions. For reactions with ethyl acetate, cyclohexylamine consistently produced lower amide yields than benzylamine (Table S1). Although cyclohexylamine is intrinsically a stronger nucleophile, its bulky cyclic structure imposes a greater steric barrier during nucleophilic attack, leading to slower reaction rates. This trend is consistent with previous studies reporting lower rate constants for cyclohexylamine (0.05 h^−1^) compared to benzylamine (0.2 h^−1^) in amide synthesis [8], and with a recent study demonstrating higher overall reactivity of benzylamine than cyclohexylamine in hydrothermal fluids [32]. Steric effects are also evident in the esters. When reacted with benzylamine, ethyl benzoate formed substantially lower amounts of amides compared to ethyl acetate, despite being the more electrophilic ester. This outcome suggests that steric hindrance around the benzyl carbonyl (and reduced accessibility of the transition state) is more limiting than electrophilicity alone. The highest amide yields were obtained from ethyl acetate and benzylamine, reflecting a combination of low steric hindrance at both the nucleophile and the ester. In contrast, no amides were detected in the reactions between esters and secondary amine, diphenylamine, which is more bulky and less nucleophilic than the primary amines. This result is consistent with other studies that showed unfavorable amide formation from secondary amines [33].

**FIGURE 3:**
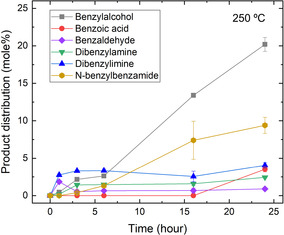
Product distribution (mole%) of hydrothermal reaction between benzylamine and ethyl benzoate at 250°C and *P*_sat_ for up to 24 h.

In addition to steric and nucleophilic effects, our results indicate that ester hydrolysis can compete directly with aminolysis under the conditions studied. For example, ethyl benzoate undergoes rapid hydrolysis (>90% conversion to benzoic acid within 1 h) (Table S2), suggesting that the hydrolysis–condensation pathway may dominate. Aside from hydrolysis, the overall amide yield was also affected by self‐reactions of benzylamine, such as substitution to form benzyl alcohol, self‐coupling to form dibenzylamine and dibenzylimine, and oxidation to form benzaldehyde (Figure 3). This consumes much of the amine available and reduces the efficiency of the amide synthesis pathway. The side products observed from the self‐reactions of benzylamine are also consistent with previous studies on deamination of benzylamine under similar hydrothermal conditions [32, 34]. However, the direct aminolysis cannot be ruled out as a contributing pathway. The relative rates of aminolysis and hydrolysis are expected to depend on substrate identity, amine concentration, water activity, and temperature. Accordingly, direct aminolysis may be more favorable under conditions that increase the encounter frequency of amine and ester, use esters with better leaving groups, or reduce the water activity.

**FIGURE 4:**
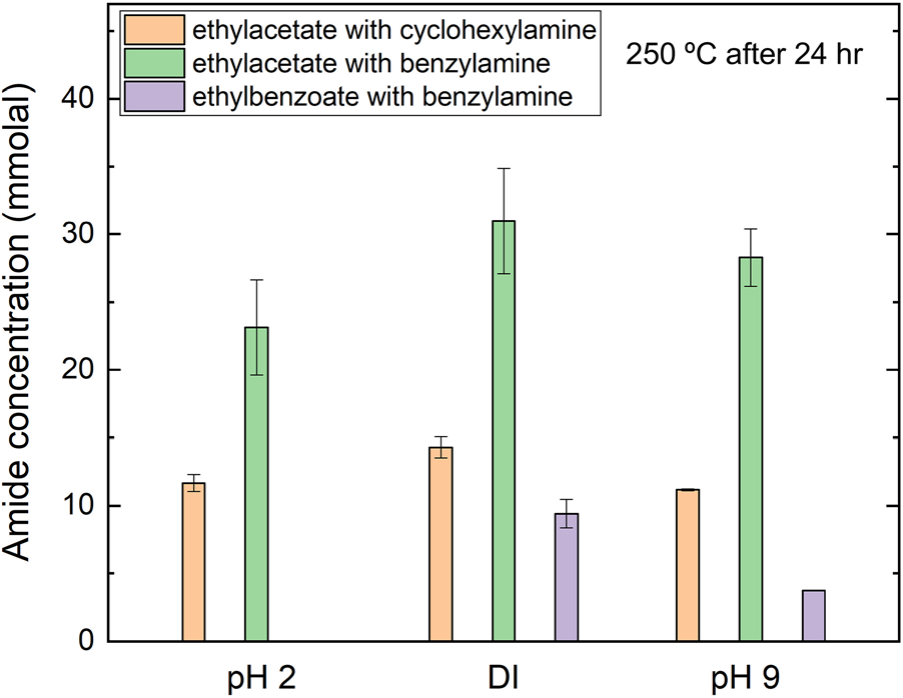
Synthesized amide concentration (mmolal) from esters and amines in acidic (pH = 2), neutral (DI), and basic (pH = 9) hydrothermal solutions at 250°C after 24 h.

The pH effect on amide synthesis was also investigated through hydrothermal experiments conducted under acidic (in situ pH of 2.1), neutral (DI), and basic (in situ pH of 9.4) conditions at 250°C and *P*_sat_ for 24 h (Table S3). At all studied pH conditions, the amide yields follow the trend of ethyl benzoate with benzylamine < ethyl acetate with cyclohexylamine < ethyl acetate with benzylamine (Figure 4). Additionally, a higher yield of amides was consistently observed in DI compared to acidic and basic conditions. Ethyl acetate and cyclohexylamine produced ≈14 mmolal N‐cyclohexylacetamide in DI, compared to 11–12 mmolal at pH 2.1 and 9.4 (Figure 4). Ethyl acetate and benzylamine yielded ≈31 mmolal N‐benzylacetamide under neutral pH, compared to 23 and 28 mmolal at pH 2.1 and 9.4, respectively. The pH effect was most pronounced for ethyl benzoate and benzylamine, with no amide formed in acidic solutions, ≈9 mmolal N‐benzylbenzamide in DI, and ≈4 mmolal amide formed in basic solutions. These results suggest that hydrothermal amide synthesis is pH‐sensitive, which is also consistent with a previous study reporting an inhibition of amide formation in highly basic (e.g., pH > 12) or acidic (e.g., pH < 2) hydrothermal solutions [8]. Under acidic conditions, primary amines such as benzylamine could predominantly exist in their protonated aminium form (R‐NH_3_^+^) [32, 34], which dramatically lowers their nucleophilicity and slows down the reaction, thereby reducing the amide yield. Formation of aminium also promotes amine self‐reactions to alcohols and diamines [32, 34], which contributes to lower amide yields. In contrast, at higher pH, more of the amines exist in their neutral or deprotonated form, which enhances the aminolysis reaction compared to acidic solutions. The results suggest that hydrothermal amide synthesis more readily occurs at nonacidic conditions.

**FIGURE 5:**
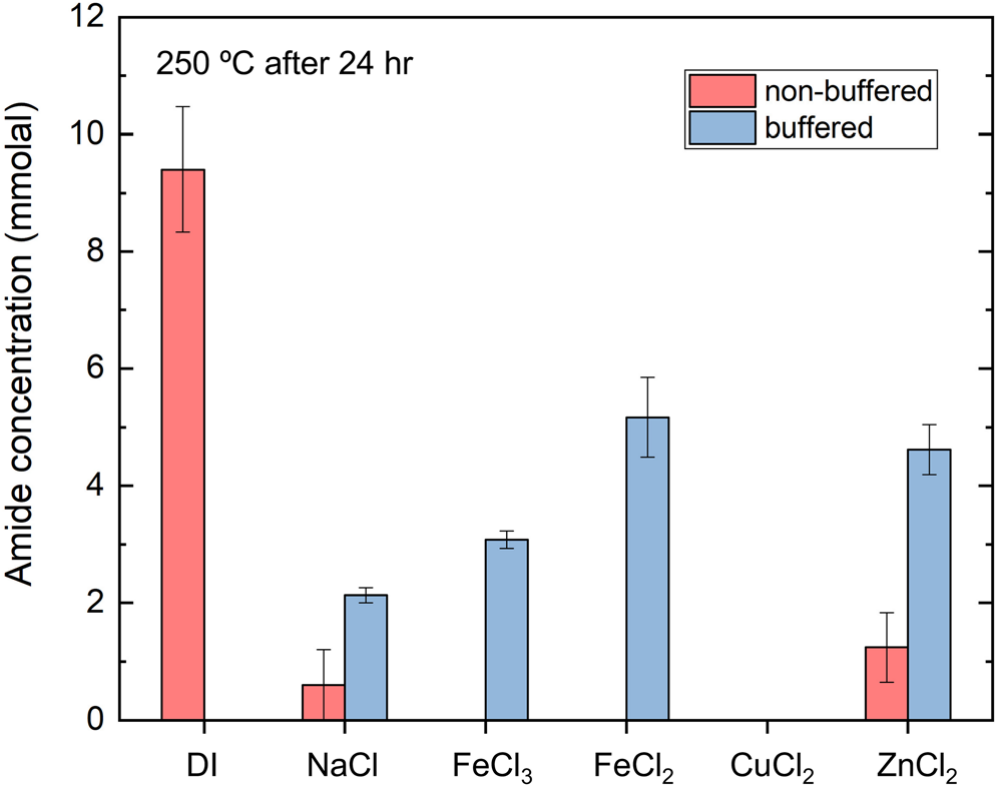
Synthesis of amide from ethyl benzoate and benzylamine in the absence and presence of metal salts under pH‐buffered and nonbuffered hydrothermal conditions of 250°C and *P*_sat_ after 24 h.

The effects of several metal salts (e.g., NaCl, FeCl_3_, FeCl_2_, CuCl_2_, and ZnCl_2_) on amide synthesis were also examined under pH‐buffered (phosphate buffer) and nonbuffered conditions (Figure 5). These metal salts were selected because they are commonly distributed in natural hydrothermal environments [35] and have been shown to significantly affect the reactivity of multiple organic functional groups in hydrothermal systems [22, 30, 31]. In the nonbuffered metal salt experiments, amide formation was significantly inhibited by all of the metal salts. Only NaCl and ZnCl_2_ allowed a small amount of amide formation (0.6 and 1.2 mmolal, respectively), while all other salts completely inhibited the amide formation (Figure 5, Table S4). Previous studies have suggested that solution pH, ionic strength, and metal salt speciation are important factors influencing the hydrothermal transformation pathways and product distribution of organic compounds [36, 37]. Among the selected metal salts, most of them are acid salts or Lewis acids. Thermodynamic calculations also demonstrate that dissolution of these metal salts can result in significant decreases in solution pH and ionic strength under the hydrothermal conditions (Table S5), which could cause a decrease in amide yields, as shown in the pH‐regulated experiments (Figure 4).

To determine whether the observed inhibition of amide synthesis was due to the change of pH in metal salt solutions, the same experiments were conducted with the metal salts in a phosphate buffer. When the metal salt solutions were adjusted to the same pH (5.6), significant recovery of amide formation was observed. NaCl, FeCl_3_, FeCl_2_, and ZnCl_2_ yielded 2–5 mmolal of amide, whereas CuCl_2_ exhibited no amide formation (Figure 5, Table S4). These results indicate that much of the inhibitory effect in the unbuffered metal salt solutions is likely pH‐driven, since many of the metal salts form acidic solutions upon dissolution. However, low amide yields were also observed in non‐acidic NaCl solutions, which implies that other factors such as ionic strength or phosphate complexation may also influence the reaction. Previous studies have shown that electrolytes can stabilize charged transition states, but higher ionic strengths can lower the self‐ionization of water, reducing the activity of H^+^ and OH^−^ ions [16, 37, 38]. Metal cations such as Zn^2+^ and Mg^2+^ are also known to facilitate the deprotonation of phosphates and form complexes with phosphate ions [39], which may further limit the amide formation.

Interestingly, CuCl_2_ is the only salt that yielded no amides in both buffered and unbuffered solutions (Figure 5). Previous studies have shown that Cu^2+^ strongly tends to form complexes with anions such as Cl^−^ and SO_4_^2−^ under hydrothermal conditions [36, 37], and Cu^2+^ is known to coordinate to phosphate groups in nucleotides [40]. Although the complexation between Cu^2+^ and anions may interfere with amide formation in hydrothermal solutions, the observed amide inhibition is more likely due to the highly oxidizing effect of Cu^2+^, which is known as a strong oxidant for organic compounds in hydrothermal systems [11, 22]. For example, Fu et al. have shown that the presence of Cu^2+^ can greatly lower the amide yield from hydrothermal condensation between benzylamines and carboxylic acids, producing oxidized products such as benzaldehyde in large quantities [22]. Consistently, in the present study, substantial amounts of benzaldehyde (20–24 mol% in CuCl_2_, compared to <3 mol% in DI, Figure 3) were also observed in the experiments of benzylamine and ethyl benzoate with Cu^2+^, which suggests a key role of Cu^2+^ in oxidizing amines and therefore preventing the amide formation. These results further suggest that metal salts may play multiple roles in driving or limiting the amide formation under hydrothermal conditions.

## 4 Conclusion

In this study, we explored amide synthesis under hydrothermal conditions, using amines and esters as the starting materials. Both aromatic and aliphatic amines and esters show the feasibility of amide synthesis. The amide yield is found to be dependent on the substrate structure, solution pH, and the presence of dissolved metal salts. Hydrolysis of esters occurs rapidly under hydrothermal conditions, which likely contributes significantly to amide formation via condensation with the resulting carboxylic acids, although direct aminolysis may still occur. The presence of metal salts can have a significant inhibition on amide formation, due to the decrease of solution pH by the dissolution of acidic salts or strong redox effects. The amide formation can also be affected by the relatively high ionic strength or the complexation with phosphate ions. Overall, our findings show how the complex interplay between organics, inorganics, and solution chemistry may influence the amide synthesis in hydrothermal systems. Future work on investigating a larger scope of reaction substrates and the effect of other Earth‐abundant materials, such as minerals, on amide hydrothermal synthesis would be beneficial.

## Supporting Information

Additional supporting information can be found online in the Supporting Information section. Supporting information, including Tables S1–S5 and Figures S1–S5, is available at https://doi.org/10.1002/open.202500508. **Supporting Fig. S1:** GC‐MS identification of N‐benzylacetamide formed from ethyl acetate and benzylamine under the hydrothermal conditions. **Supporting Fig. S2:** GC‐MS identification of N‐benzylbenzamide formed from ethyl benzoate and benzylamine under the hydrothermal conditions. **Supporting Fig. S3:** GC‐MS identification of N‐cyclohexylacetamide formed from ethyl acetate and cyclohexylamine under the hydrothermal conditions. **Supporting Fig. S4:** ^1^H NMR spectra of N‐benzylacetamide synthesized from ethyl acetate and benzylamine under the hydrothermal conditions. **Supporting Fig. S5:** ^1^H NMR spectra of N‐benzylbenzamide synthesized from ethyl acetate and benzylamine under the hydrothermal conditions. **Supporting Table S1:** Amide concentrations (mmolal) and yields (%) from hydrothermal reactions between different esters (0.2 molal) and amines (0.1 molal) in DI water at 250°C and *P*_sat_ after 6 and 24 h. **Supporting Table S2:** Hydrolysis of ethyl benzoate (0.2 molal) to form benzoic acid in DI at 250°C and *P*_sat_ after 1, 6, and 24 h. **Supporting Table S3:** Amide concentrations (mmolal) from hydrothermal reactions between esters (0.2 molal) and amines (0.1 molal) in acidic (pH 2.1), neutral (DI), and basic (pH 9.4) water at 250°C and *P*_sat_ after 24 h. **Supporting Table S4:** Formation of amides (mmolal) from hydrothermal reactions between ethyl benzoate (0.2 molal) and benzylamine (0.1 molal) in pH‐buffered (pH 5.6) and non‐buffered metal salt solutions at 250°C and *P*_sat_ after 24 h. **Supporting Table S5:** Thermodynamic calculations of in‐situ pH and ionic strength in the acidic (with HCl), neutral (DI), basic (with NaOH), and metal salt solutions (0.1 molal) under the hydrothermal conditions (250°C and *P*_sat_).

## Funding

National Science Foundation (OCE‐2042213).

## Conflicts of Interest

The authors declare no conflicts of interest in this paper.

## Supporting information

Supplementary Material
