# Supplementary material for: Synthesis of Amides from Amines and Esters Under Hydrothermal Conditions
Source: ChemistryOpen. 2026 Feb 5;15(2):e202500508. doi: 10.1002/open.202500508 (PMC12875678; doi:10.1002/open.202500508)

Supporting Information

Synthesis of Amides from Esters and Amines under Hydrothermal Conditions

Prince Antwi Brown[1], Alexandria Aspin[1], and Ziming Yang[1*]

[1]Department of Chemistry, Oakland University, Rochester, MI 48309, USA.

Corresponding author: Ziming Yang (zimingyang@oakland.edu)

Table S1: Amide concentrations (mmolal) and yields (%) from hydrothermal reactions between different esters (0.2 molal) and amines (0.1 molal) in DI water at 250 °C and P_{sat} after 6 and 24 hours. n.d. means not detected. Uncertainties represent standard deviation from replicate experiments.

**Reaction**	**Amide product**	**Amide concentration after 6 h (mmolal)**	**Amide yield (%) after 6 h**	**Amide concentration after 24 h (mmolal)**	**Amide yield (%) after 24 h**
Ethyl acetate + benzylamine	N-Benzylacetamide	13.1	13.1%	30.9 ± 3.8	30.9%
Ethyl benzoate + benzylamine	N-Benzylbenzamide	1.3 ± 0.1	1.3%	9.4 ± 1.1	9.4%
Ethyl acetate + cyclohexylamine	N-Cyclohexylacetamide	11.8	11.8%	14.2 ± 0.7	14.2%
Ethyl benzoate + cyclohexylamine	N-Cyclohexylbenzamide	n.d.	n.d.	n.d.	n.d.
Ethyl acetate + diphenylamine	N, N-Diphenylacetamide	n.d.	n.d.	n.d.	n.d.
Ethyl benzoate + diphenylamine	N, N-Diphenylbenzamide	n.d.	n.d.	n.d.	n.d.

Table S2. Hydrolysis of ethyl benzoate (0.2 molal) to form benzoic acid in DI at 250 °C and P_{sat} after 1, 6, and 24 hours. Uncertainties represent standard deviation from replicate experiments.

Time (hr)	Ethyl benzoate concentration (mmolal)	Benzoic acid concentration (mmolal)
1	12.7 ± 2.8	184.0 ±0.7
6	2.4	190.8
24	2.4 ± 0.1	199.4 ± 2.4

Table S3. Amide concentrations (mmolal) from hydrothermal reactions between esters (0.2 molal) and amines (0.1 molal) in acidic (pH 2.1), neutral (DI), and basic (pH 9.4) water at 250 °C and P_{sat} after 24 hours. n.d. means not detected. Uncertainties represent standard deviation from replicate experiments.

**Reaction**	**Amide product**	**Amide concentration (mmolal) at pH 2.1**	**Amide concentration (mmolal) at neutral pH (DI)**	**Amide concentration (mmolal) at pH 9.4**
Ethyl acetate + benzylamine	N-Benzylacetamide	23.1 ± 3.5	30.9 ± 3.8	28.2 ± 2.1
Ethyl benzoate + benzylamine	N-Benzylbenzamide	n.d.	9.4 ± 1.1	3.7 ± 0.1
Ethyl acetate + cyclohexylamine	N-Cyclohexylacetamide	11.6 ± 0.6	14.2 ± 0.7	11.2 ± 0.1

Table S4. Formation of amides (mmolal) from hydrothermal reactions between ethyl benzoate (0.2 molal) and benzylamine (0.1 molal) in pH-buffered (pH 5.6) and non-buffered metal salt solutions at 250 °C and P_{sat} after 24 hours. n.d. means not detected. Uncertainties represent standard deviation from replicate experiments.

**Salts**	**Amide concentration (mmolal) in pH buffered solutions**	**Amide concentration (mmolal) in non-pH buffered solutions**
None		9.4 ± 1.1
NaCl (0.1 molal)	2.1 ± 0.1	0.6 ± 0.6
$FeCl_3$ (0.1 molal)	3.1 ± 0.2	n.d.
$FeCl_2$ (0.1 molal)	5.2 ± 0.7	n.d.
$CuCl_2$ (0.1 molal)	n.d.	n.d.
$ZnCl_2$ (0.1 molal)	4.6 ± 0.4	1.2 ± 0.6

Table S5. Thermodynamic calculations of in-situ pH and ionic strength in the acidic (with HCl), neutral (DI), basic (with NaOH), and metal salt solutions (0.1 molal) under the hydrothermal conditions (250 °C and P_{sat}).

Solution	pH at 22 °C	In-situ pH at 250 °C	Ionic strength at 22 °C (mmolal)	Ionic strength at 250 °C (mmolal)
DI	5.6	5.5	0	0
HCl	2.1	2.1	4.2	4.2
NaOH	12.3	9.4	1.1	1.1
$CuCl_2$	3.2	3.1	277.8	149.6
$FeCl_3$	2.1	1.2	589.7	321.8
$ZnCl_2$	5.7	3.7	281.0	90.5
$FeCl_2$	3.4	3.4	296.7	204.0
NaCl	5.4	5.3	99.0	90.0

Figure S1. GC-MS identification of N-benzylacetamide formed from ethyl acetate and benzylamine under the hydrothermal conditions.

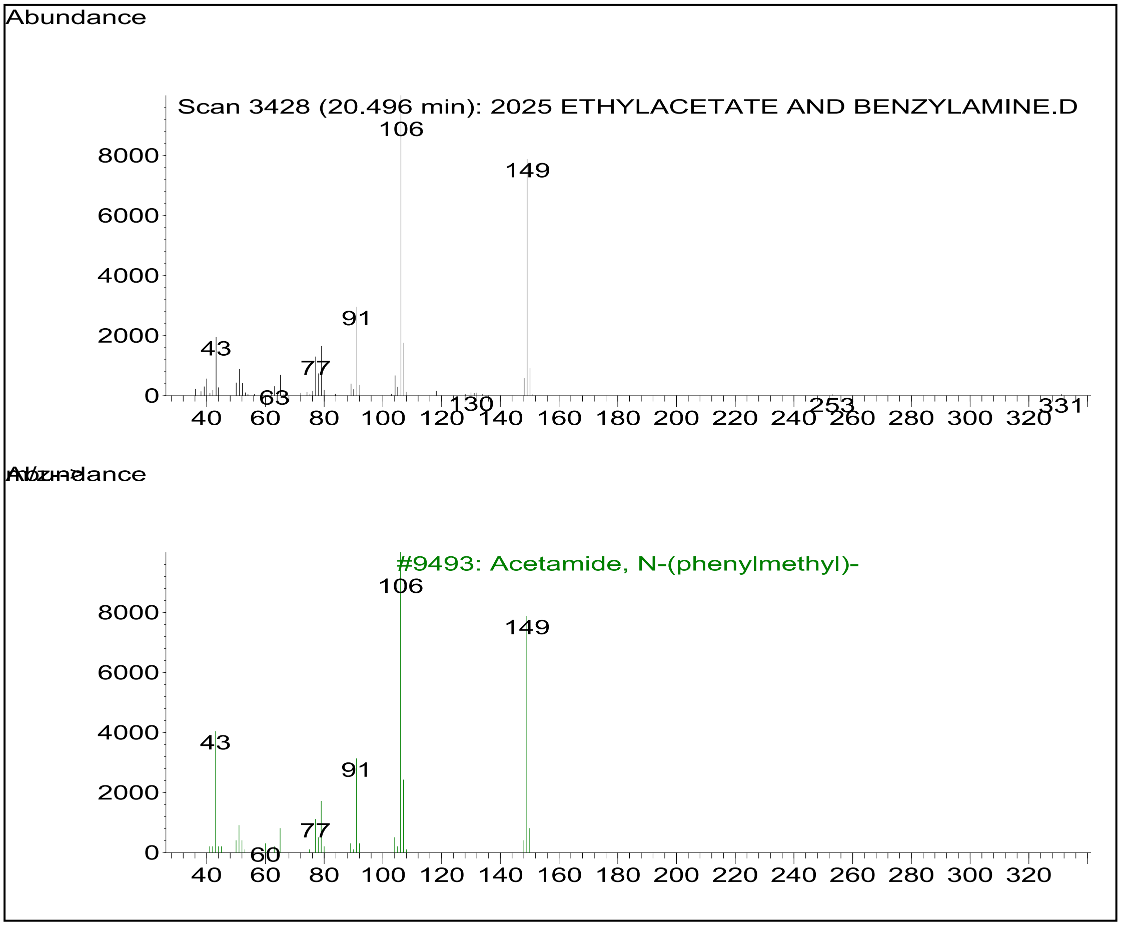

Figure S2. GC-MS identification of N-benzylbenzamide formed from ethyl benzoate and benzylamine under the hydrothermal conditions.

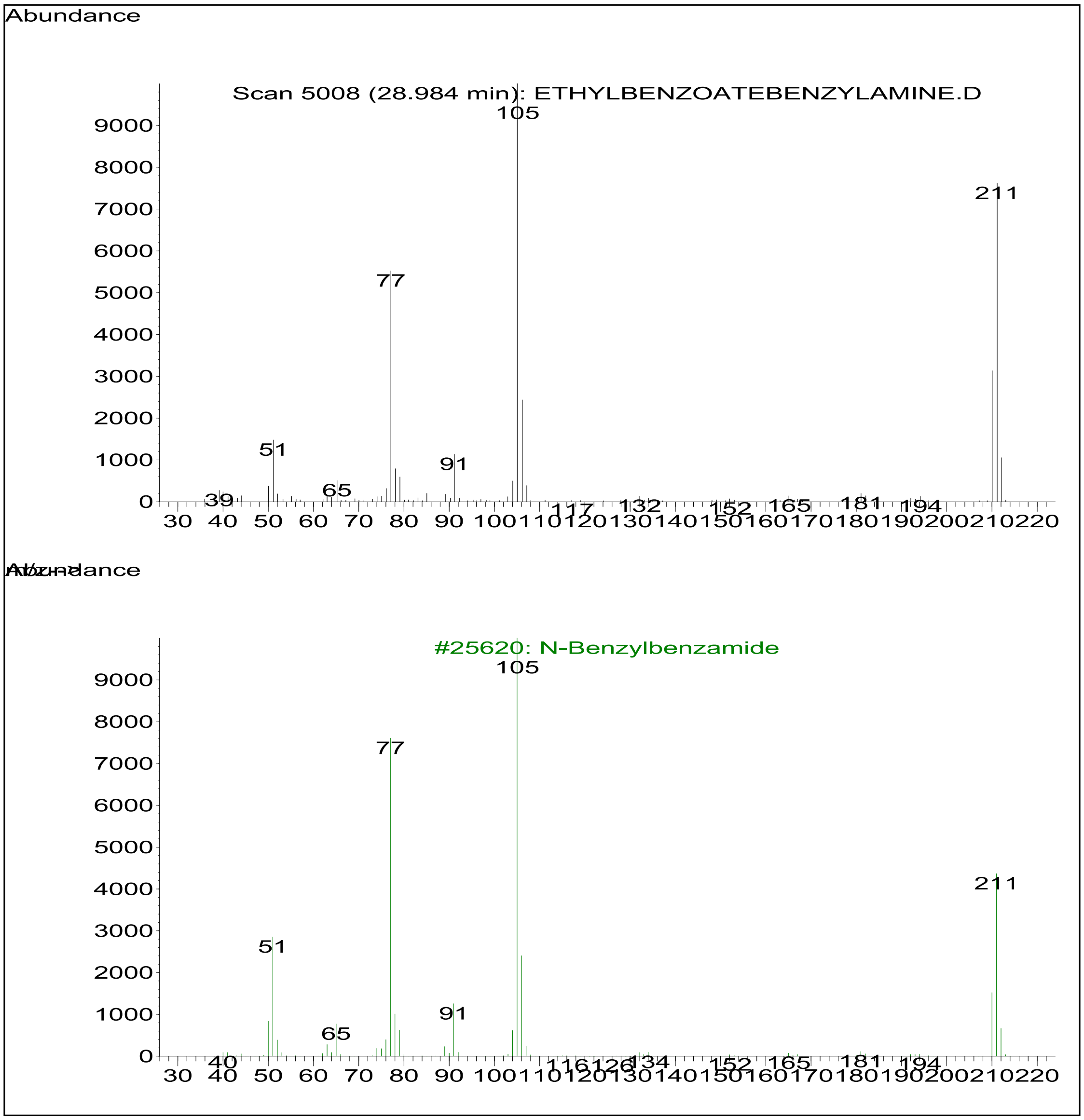

Figure S3. GC-MS identification of N-cyclohexylacetamide formed from ethyl acetate and cyclohexylamine under the hydrothermal conditions.

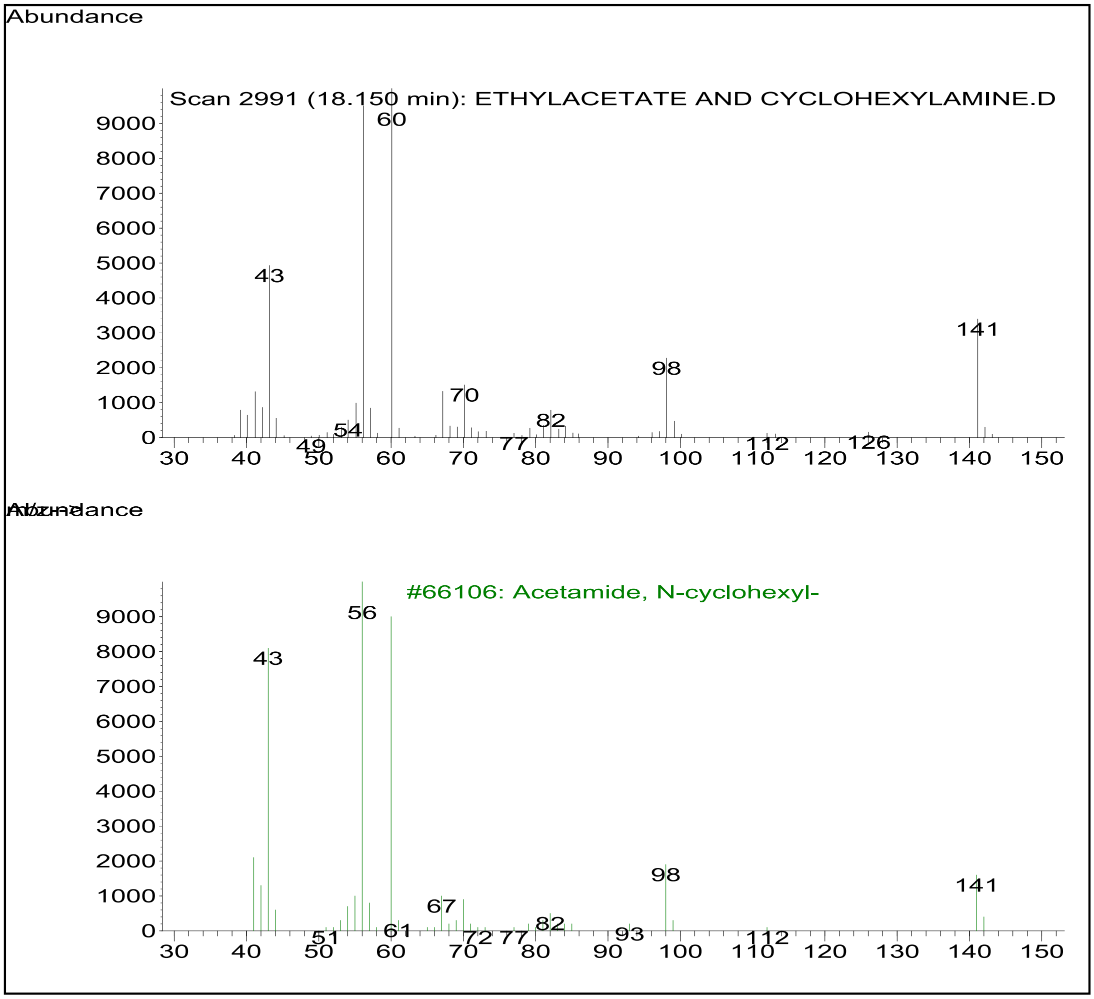

Figure S4. ^{1}H NMR spectra of N-benzylacetamide synthesized from ethyl acetate and benzylamine under the hydrothermal conditions.

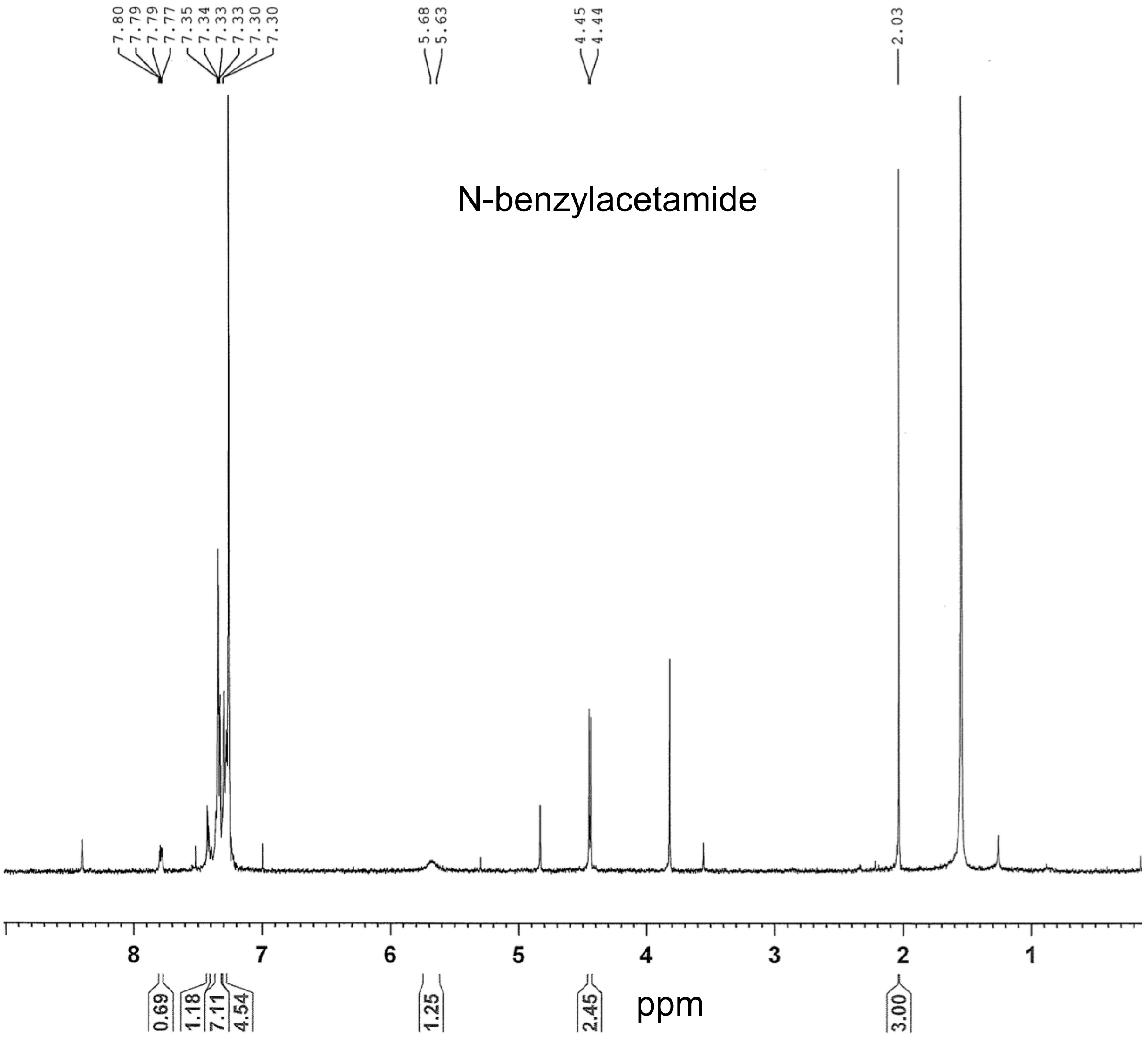

Figure S5. ^{1}H NMR spectra of N-benzylbenzamide synthesized from ethyl acetate and benzylamine under the hydrothermal conditions.

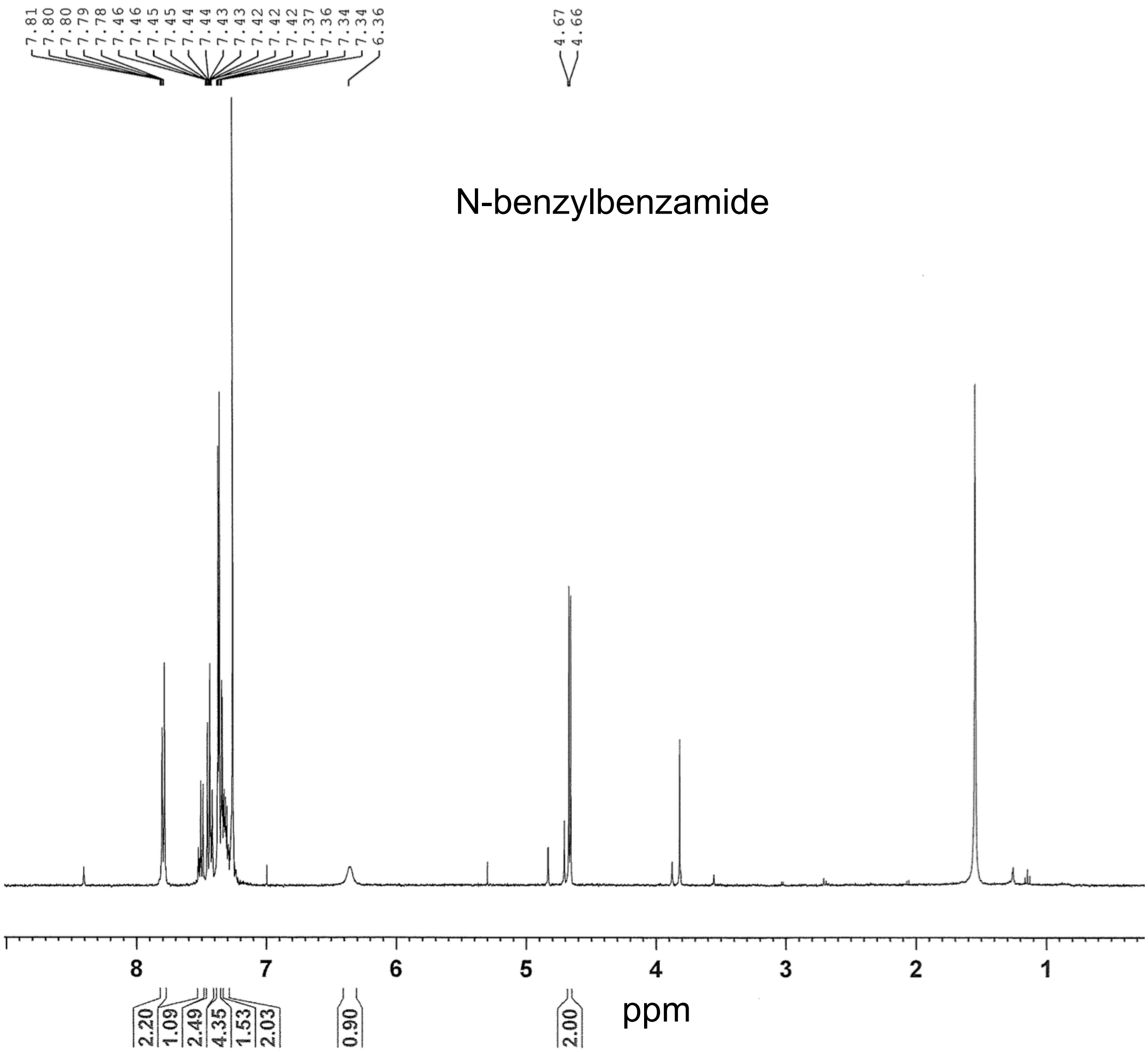

Supplement: Supplementary file 1 — Supplementary Material [file OPEN-15-e202500508-s001.pdf]
